# An Update on Canine Adenovirus Type 2 and Its Vectors

**DOI:** 10.3390/v2092134

**Published:** 2010-09-27

**Authors:** Thierry Bru, Sara Salinas, Eric J. Kremer

**Affiliations:** 1 Institut de Génétique Moléculaire de Montpellier, CNRS UMR 5535, 1919 Route de Mende Montpellier, 34293 France; E-Mails: thierry.bru@igmm.cnrs.fr (T.B.); sara.salinas@igmm.cnrs.fr (S.S.); 2 Université de Montpellier I, 5 Bd Henri IV, 34000 Montpellier, France; 3 Université de Montpellier II, place Eugène Bataillon, 34090 Montpellier, France

**Keywords:** canine adenovirus, vectors, CAV-2, CAR, gene therapy, neurons, immunity, retrograde transport, neurodegenerative diseases, vaccines

## Abstract

Adenovirus vectors have significant potential for long- or short-term gene transfer. Preclinical and clinical studies using human derived adenoviruses (HAd) have demonstrated the feasibility of flexible hybrid vector designs, robust expression and induction of protective immunity. However, clinical use of HAd vectors can, under some conditions, be limited by pre-existing vector immunity. Pre-existing humoral and cellular anti-capsid immunity limits the efficacy and duration of transgene expression and is poorly circumvented by injections of larger doses and immuno-suppressing drugs. This review updates canine adenovirus serotype 2 (CAV-2, also known as CAdV-2) biology and gives an overview of the generation of early region 1 (E1)-deleted to helper-dependent (HD) CAV-2 vectors. We also summarize the essential characteristics concerning their interaction with the anti-HAd memory immune responses in humans, the preferential transduction of neurons, and its high level of retrograde axonal transport in the central and peripheral nervous system. CAV-2 vectors are particularly interesting tools to study the pathophysiology and potential treatment of neurodegenerative diseases, as anti-tumoral and anti-viral vaccines, tracer of synaptic junctions, oncolytic virus and as a platform to generate chimeric vectors.

## 1. Introduction

*Adenoviridae* infect a variety of vertebrates including mammals, fish, birds and reptiles [1]. More than 50 human adenoviruses (HAd) serotypes have been identified. A handful of these are extensively studied because of their tendency to induce respiratory, ocular, and enteric infections in immunocompetent individuals as well as morbidity and mortality in severely immunodeficient patients [2–6]. Early generations of HAd vectors, primarily ΔE1/E3, are often efficient for a limited duration in laboratory animals that have never been exposed to the wild type viruses. However, subsequent deliveries lead to inefficient gene transfer. The poor efficacy and limited duration are mainly due to neutralizing antibodies that prevent transduction and to CD8+ and CD4+ cytotoxic T lymphocytes (CTLs) that destroy transduced cells. This memory immune response can also be problematic for clinical use of vectors. Decades ago, epidemiology studies reported that the majority of us had been exposed to multiple adenovirus serotypes by the time we were 10 years old [7–9]. This leads to a cross-reacting humoral, as well as cellular response against many serotypes [10–12]. To reduce or circumvent these drawbacks, strategies including the induction of immunotolerance, immunosuppression, or chemical and genetic modifications on viral vectors have been considered. In the early 1990s, we and others considered the possibility of using nonhuman vectors. We began designing canine adenovirus serotype 2 (CAV-2) vectors [13–17], while others were generating vectors from ovine, bovine, chimpanzee, simian or porcine [18–22]. We, and surely others, hypothesized that vectors derived from nonhuman adenoviruses would be more clinically useful than those from HAds based, in part, on the potential lack of immunological memory [23]. In spite of the approx 100,000 years of cohabitation of humans with dogs, CAV-2 has not been known to cross the species barrier and is unable to replicate in human cells. Therefore, our hope was to keep the numerous advantages associated with HAds vectors, while bypassing some of their clinical disadvantages.

## 2. Vectors

Plasmids harboring first-generation CAV-2 vector genomes are created by *in vivo* homologous recombination in *E. coli* BJ5183 between pTG5412, which contains the full-length CAV-2 genome, and a fragment of DNA containing the inverted terminal repeat (ITR), a transgene expression cassette and the CAV-2 protein IX and E2B coding regions [15]. This strategy is similar to that described initially by Chartier *et al.* [24]. Recombination gives rise to the formation of a plasmid harboring the E1-deleted CAV-2 genome. To generate the vector, CAV-2 E1-expressing cell lines derived from dog kidney cells such as DKCre and DKZeo cells are transfected and the vector serial amplified [15,25].

The latest and most efficient adenovirus vectors for long-term gene transfer are referred to as “helper dependent” (HD) and are deleted in all viral coding regions [17,26–29]. To generate the HD CAV-2 vector, an initial subcloning step consisted of the insertion of the transgene expression cassette in a plasmid called “pGut” containing a CAV-2 ITR and an overlapping region with a plasmid called pEJK25 (see below) [17]. Once a modified pGut is generated, it is linearized and subjected to homologous recombination in *E. coli* BJ5183 with common regions present on pEJK25, which contains two ITR’s, a packaging signal and 25 kb of stuffer sequence. Like HD HAd vectors, HD CAV-2 vectors are amplified by the co-replication and preferential packaging via a Cre recombinase induced-packaging-defective helper vector which supplies the regulatory and structural proteins in *trans* (see review by Philip Ng in this issue). HD vectors improved the efficiency and duration of transgene expression mainly due to the elimination of the adaptive cell-mediated immune response in immunologically naïve animals. HD CAV-2 vectors have a high cloning capacity (∼30 kb), and allowed long-term (>1 year) transgene expression in the immunocompetent rat brain without immunosuppression [17].

## 3. CAV-2 tropism

Following the generation of CAV-2 vectors, the next challenge was to define what they would transduce *in vitro* and *in vivo*. Similar to HAd5, CAV-2 causes an upper respiratory track disease in dogs called kennel cough [30]. Moreover, anti-CAV-2 antibodies have also been found in foxes, bears and pandas [31], suggesting that either there is a cross-reacting humoral immunity to Ads that infect these species or that CAV-2 also naturally propagates in other hosts. Interestingly, CAV-2 has also been detected in the CNS of dogs and foxes.

The gene transfer field long ago moved away from the dogma that vector tropism is restricted to tissues that manifest disease symptoms in wild type infections. Via specific delivery routes (intravenous, intranasal, intracerebral, intramuscular, *etc.*), one can partially dictate the population of cells available for transduction, and therefore the tropism. Using intracerebral and intramuscular injections, we found that CAV-2 vectors preferentially transduce neurons [16]. When CAV-2 vectors were instilled in the rat olfactory cavity, the sensory olfactory neurons, in contrast to the columnar epithelial cells, were preferentially transduced. Injection in the hind leg muscle (gastrocnemius) and tibias anterior in newborn mice showed a poor level of transduction of the myofibers, but a surprising level of specificity for the innervating motor neurons via retrograde axonal transport. Similarly, following injection into the highly innervated diaphragm, an insignificant level of muscle cells were transduced but a significant number of neuromuscular junctions were GFP^+^, demonstrating again the transduction of motor neurons and axonal retrograde transport of CAV-2.

**Figure 1.**
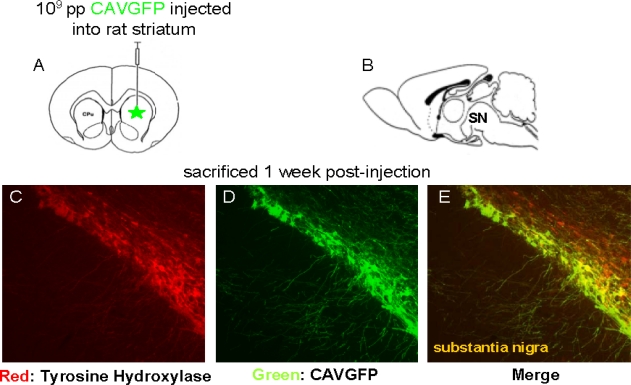
Stereotactic injection of a CAV-2 vector expressing GFP in the rat striatum. **(A**–**B)** Schematic inserts show respectively midsagittal and frontal cross sections of the rat brain and the location of the injection site (striatum) relative to the location of the transduced cells (substantia nigra (denoted **SN**)). **(C)** Immunolabeling for tyrosine hydroxylase (TH) expressed in the substantia nigra (red). **(D)** Retrograde axonal transport and expression in the vector-transduced neurons (green) in the substantia nigra. **(E)** Colocalization of GFP with TH.

When injected into the brain parenchyma, CAV-2 vectors preferentially transduced neurons at the site of injection, as well as the neurons that project into this structure. Following injection in the striatum, the dopaminergic neurons of the substantia nigra pars compacta, neurons in layer IV of the neocortex and thalamus were transduced. We found that, via a single injection of 10^9^ pp in the striatum of rats, CAV-2 vectors can transduce >70% of the dopaminergic neurons in substantia nigra pars compacta (Figure 1).

Not surprisingly, little was known concerning the determinants for CAV-2 long-range axonal transport. Axonal transport is essential for neuronal homeostasis as its impairment is often associated with neurodegenerative disorders. Some viruses, including rabies, herpes simplex type I (HSV-1) and poliovirus, as well as bacterial toxins such as tetanus toxin, use this process to access the nervous system [32]. These microbial agents can enter at nerve terminal situated in the periphery and use axonal transport to access the central and peripheral nervous systems. Notably, different mechanisms of axonal transport have been described: direct interaction with molecular motors for HSV-1 [33] *versus* endosomal trafficking for poliovirus [34] and tetanus toxin.

**Figure 2.**
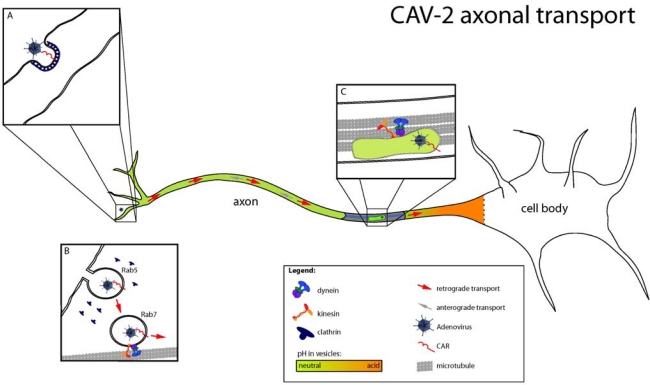
Different steps of CAV-2 axonal transport. **(A)** After binding to CAR at the cell surface, CAV-2 is internalized in clathrin-coated pits. **(B)** During entry, maturation of CAV-2 containing vesicles occurs, with a shift from Rab5 to Rab7 endosomes that will eventually lead to CAV-2 axonal transport. Despite Rab7 maturation, the pH of axonal endosomes stays neutral, and CAV-2 remains inside vesicles during transport. **(C)** CAV-2 traffic involves dynein and kinesin, with a bias for the retrograde direction. CAR is still found in CAV-2 positive vesicles. (Image modified from Henaff and Salinas, *Virulence* [36]).

Using a combination of ultrastructural analyses, live-cell imaging and cell biology approaches, we recently characterized the molecular mechanisms regulating CAV-2 entry and transport in primary rodent motor neurons [35,36]. CAV-2 is retrogradely transported in motor neuronal axons of the sciatic nerve after intramuscular injection in hind leg of mice or primates. *In vitro*, we found that CAV-2 trafficking was bidirectional with a preferential retrograde transport, suggesting coordination between molecular motors of different polarities. This was confirmed by genetic inhibition of dynein (responsible for the retrograde transport from nerve terminals to cell bodies) and kinesin (responsible for the anterograde transport from cell bodies to nerve terminals), which led to a similar impairment of CAV-2 axonal transport. Ultrastructural analyses and live-cell imaging using fluorescent markers of endocytic organelles showed that CAV-2 transport was occurring inside endosomes, which matured from Rab5 to Rab7 during entry and transport (Figure 2). In epithelial cells, some serotypes of adenoviruses escape from Rab5 early endosomes due to acidification and conformational changes in the viral capsid that trigger membrane lysis [37,38]. Once in the cytoplasm, viruses may recruit dynein directly through the hexon protein to be transported to the nuclei [39]. To understand why CAV-2 is transported inside endosomes during its transport, we measured the pH of axonal endosomes using carboxyfluorescein-coupled virions. We found that CAV-2 was transported in endocytic organelles whose lumens were close to pH neutral. This vesicle likely allows long-range transport in a “protective” environment, precluding degradation or pH-induced conformational changes of the capsid and endosomal escape. Interestingly, this vesicular pathway is also used by tetanus toxin, poliovirus, and endogenous molecules such as neurotrophins and their receptors, suggesting that CAV-2 is taking advantage of an endogenous pathway crucial in neuronal differentiation and survival [32].

To gain further insight into how CAV-2 accessed this endocytic trafficking pathway, we characterized the role of Coxsackievirus and adenovirus receptor (CAR) [15,40] in the entry and transport of CAV-2. Whereas CAR’s function is partially described in tight-junctions of epithelia, no clear role in neurons has been described [41]. Notably, CAR is highly expressed during development of the nervous system and is also found in the adult nervous system [41–43]. Using competition experiments, we found that CAR was necessary for CAV-2 binding to the neuronal membrane and was also co-internalized with virions. Using fluorescently labeled CAV-2 fiber knob, we showed that CAR could enter and be transported bi-directionally in axons similar to CAV-2. These unsuspected vesicular dynamics of CAR opens numerous questions regarding its role in neuronal adhesion and possibly axonal transport. Together, these data suggest that CAV-2’s ability to infect neurons and be retrogradely transported could be due to the innate ability of CAR to access this trafficking pathway.

These data do not mean that CAV-2 vectors transduce exclusively neurons. Not surprisingly, CAV-2 also efficiently transduce epithelia of the upper and lower respiratory tract and alveolar cells after deep nasal instillation in mice [15,44]. In addition, one must keep in mind possible differences between “*mice and men*”. Although CAV-2 vectors may transduce certain murine tissues, this does not, *a priori*, mimic the efficiency of transduction in the clinic. We also assayed CAV-2 transduction *ex vivo* in temporal lobectomy specimens from epileptic patients and found preferential infection of neurons was not specific to rodents [16].

## 4. CAV-2 receptors

Direct observation of CAV-2 transduction was the first step to determine vector tropism *in vitro* and *in vivo*. Then, it was necessary to understand how CAV-2 interacts with cells at the molecular level.

Adenovirus entry into the cytoplasm can be functionally divided into attachment, internalization and permeabilization of the endosomal membrane. CAV-2, like all other adenoviruses, has an icosahedral capsid with the external surface composed mainly of hexon, penton base, and fiber. The fiber is an elongated thread-like molecule that projects from the penton base and initiates binding to the cellular surface. The C-terminal fiber knob domain of many Ads, such as HAd2/5, attaches to CAR [45–47]. Attachment of the virus is followed by internalization and permeabilization in clathrin-coated pits implicating dynamin [48], and α_v_β_5_ and α_v_β_3_ integrins [49]. The α_v_ integrins recognize a conserved Arg-Gly-Asp (RGD) motif found in the adenovirus penton base and some extracellular matrix proteins. The three-dimensional structure of a recombinant soluble α_v_β_5_ integrin bound to the penton base of HAd2 revealed a 20-Å RGD-binding cleft in the globular domain [50]. The crystal structure of the adenovirus fiber knob in complex with CAR [41] and a mutational analysis [51] identified several amino acids in the knob of some adenoviruses that are critical for CAR binding [52,53].

**Figure 3.**
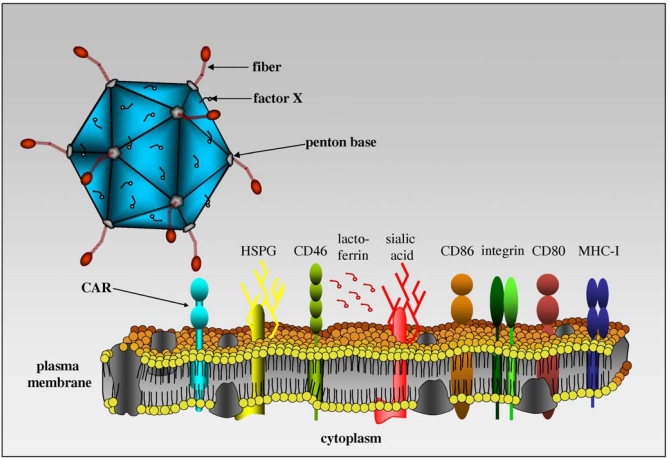
An overview of molecules involved in adenovirus binding. Adenoviruses can attach to CAR, sialic acid, CD46, CD86, CD80, MHC-I and heparin sulfate proteoglycan (HSPG) depending on the serotype. Some HAds also use coagulation factors and lactoferrin as bridges to attach to cells. To the best of our knowledge, CAR is the only receptor for CAV-2. (Image modified from Kremer, *Blood* [60]).

Our *in vitro* and *in vivo* data demonstrated that CAV-2 and HAd2/5 had overlapping, but distinct, tropism [15]. Essentially, we found that any cell or tissue that can be transduced by CAV-2 can also be transduced by HAd5, but not *vice versa*. Notably, cells expressing CAR could be transduced by CAV-2. CAR-negative cells but α_M_β_2_ integrins and/or the heavy chain of MHC-I positive could not be transduced [40,54] (Figure 3). Interestingly, the CAV-2 capsid does not contain an integrin-interacting motif - in particular the RGD motif in the penton base loop [40,55]. Using radiolabeled CAV-2, we found that CAV-2 binds to CAR, and HAd5 fiber partially blocked attachment, suggesting that CAV-2 bound CAR at or near the same epitope. Preliminary data also suggested that CAV-2 does not interact with coagulation factor [56], lactoferrin [57] and CD46 [58,59].

A comparison of the three-dimensional structures of CAV-2 and HAd5 capsid allowed us to identify differences between these two viruses. We found that CAV-2 capsid has a smoother structure than human serotypes. Many of the external loops found in the HAd5 penton base and the hexon, against which the antibody response is directed, are shorter or absent in CAV-2, which could explain the paucity of cross-reacting human neutralizing antibodies [61,62]. On the other hand, the fiber appeared to be more complex. The CAV-2 fiber shaft is similar in length to that of HAd2, with a lefthanded triple helical structure composed of β-strands interspersed with extended loops which contain 18.5 repeats with between 15 and 19 amino acids per repeat. A key difference may be the presence of two bends in the shaft [61]. Another major difference in the 3D structure of these viruses is located in protein IX. Its C-terminal part is in a different position, creating an antenna sticking out of the CAV-2 capsid. Protein IX could be a site to position additional protein domains for specific interaction with host cells.

## 5. Induced and crossreacting pre-existing immunity in humans

Twenty years ago, our initial reason to generate a nonhuman adenovirus vector was to avoid the inhibitory effects of the pre-existing humoral immunity readily found against multiple HAds serotypes, as well as to avoid the re-stimulation of memory CTLs. Due to multiple infections during childhood, most of us (>85%) possess relatively high circulating Abs against several HAd serotypes [8,63]. Antiadenovirus antibodies can recognize many capsid proteins and, in particular, hexon, penton base, and fiber proteins [64,65]. Although the neutralization activity is often described as serotype specific [66] and age-related, cross-reacting nonneutralizing anti-HAd Abs also exist. Although there are ways to eliminate anti-capsid antibodies from serum, we would argue that a limited number of interventions to treat a patient should improve the chance for an encouraging outcome. In addition, transient depletion of antiviral antibodies from a patient could put them at risk for HAd disease from latent HAds [67]. Following the generation of CAV-2 vector, we quantified neutralizing Abs (NAbs) in serum from blood bank donors. We found that sera from approximately 98% of a random cohort did not contain significant titers of neutralizing anti-CAV-2 Abs [15]. These data supported the potential clinical use of CAV-2 because the donors were infected with multiple human serotypes, developed complex and varied responses and had different genetic background. Interestingly, similar observations have been made with ovine (OAd), bovine (BAd) and porcine (PAd) adenoviruses showing that anti-OAd, anti-BAd-3 and -PAd-3 NAbs were rare in the human population but that pre-existing humoral and cellular immunity could cross-react [10,68–71]. In some case, *in vitro* neutralization assays may not reliably predict the effect of virus-specific antibodies *in vivo*. Recently, assays compared the neutralization effect of human antibodies directed against wild type chimpanzee adenovirus 68 (AdC68) [72]. Human anti-HAds antibodies failed to neutralize a mutant form of AdC68 *in vitro*, containing a 3-amino acid mutation within the major neutralization site, but impaired the vector’s capacity to transduce cells and to stimulate a transgene product-specific CD8^+^ T-cells response *in vivo*.

NAbs are only one obstacle to efficient gene transfer. Another aspect of the pre-existing immunity includes the cellular response, in particular the long-lived proliferative CD4^+^ memory cells (T_M_). We and others have questioned the clinical potential of serotype switching of HAd vectors because a HAd5-induced cytotoxic T_M_ can lyse autologous cells infected with other HAd species [73]. We predicted [23] that a T_M_ response against virion proteins, which would be poorly blunted by many immunosuppressive drugs [74], would lead to deleterious side effects in some patients [14]. We assayed CAV-2-induced human T_M_ proliferation and activation [75]. Fewer than half of the cohort harbored proliferating CD4^+^ T_M_ directed against the CAV-2 virion proteins (*versus* >85% against HAd5 vectors). Furthermore, the CAV-2 responders had a 17-fold lower activation than the HAd5 vector responders and no CD8^+^ T_M_ were detected in any donors.

In immunologically naïve rodents, CNS and respiratory tract delivery of ΔE1 CAV-2 vectors were less immunogenic than ΔE1/E3 HAd5 vectors and induced fewer infiltrating CD4^+^ and CD8^+^ cells at an equivalent number of injected particles [17,44]. Following intranasal instillation in mice, CAV-2 vectors also led to a lower level of TNF-α secretion than HAd5 vectors [44]. These data could be a result of a potential lack of capture, presentation and maturation of dendritic cells (DC) by CAV-2 [76].

Another issue that we explored was the direct interaction of the CAV-2 capsid with DC, which play a pivotal role in orchestrating and bridging innate, adaptive, and memory immunity [77,78]. Novel subtypes of DCs are also continuously being identified, ranging from Langerhans cells (myeloid) to plasmacytoïds (lymphoid) [79]. Each subtype can be artificially divided into immature or mature, which are characterized by phenotypically and functionally distinct characteristics. Immature DC sense their environment via non-specific phagocytosis and detect pathogens via evolutionarily conserved pattern recognition receptors, such as Toll-like receptors that recognize conserved microbeassociated molecules called “pathogen-associated molecular pattern” [80]. Activation promotes DC maturation, which results in the loss of their ability to take up antigens, change their morphology, and migrate towards the lymphoid compartments. Once there, matured DC are primed for antigen-specific naïve T-cell presentation and stimulation via the expression of major histocompatibility complex (MHC) class I/II and costimulatory molecules [81].

In the context of assessing the clinical relevance of CAV-2 vectors, we assayed their effect on human monocyte-derived DC (MoDC). Using a mix of functional and phenotypical assays, we found that, in contrast to the HAd5-based vectors, CAV-2 poorly transduced DC, provoked minimal upregulation of major histocompatibility complex class I/II and costimulatory molecules (CD40, CD80, and CD86), and induced negligible morphological changes indicative of DC maturation [76]. We also tested functional criteria for vector-induced MoDC maturation: reduction of antigen uptake, pertinent cytokine secretion (TNF-α, interleukin (IL)-10, IL-12, and type 1 interferon (IFN)). Again, in contrast to the three HAd5-based capsids, CAV-2 poorly induced functional characteristics of DC maturation. These results provided a partial explanation for the reductive adaptive immune response against CAV-2 vectors in rodents: poor DC transduction and maturation lead to a lower adaptive response in naïve hosts.

Together, these results showed that the CAV-2 vectors generated rare events of proliferation, activation or differentiation of T cells and also poorly transduced or induced the functional maturation of dendritic cells [10]. This may make CAV-2 vectors safer and more clinically applicable when longterm transgene expression is needed. Lack of vector-induced DC maturation may also limit an adaptive immune response following *in vivo* gene transfer. The lack of DC maturation was also seen when CAV-2 was incubated with human sera containing antibodies that recognize CAV-2 [82].

## 6. Potential uses

The preferential transduction of neurons and the high level of retrograde transport make CAV-2 vectors ideal tools to study the pathophysiology of many neurodegenerative disorders and to map complex neuronal networks *in vivo*. CAV-2 vectors are used in several studies to transfer genes encoding transcription factors, dominant negative mutants, constitutively activated kinases, *etc.*, into neurons *in vitro* and *in vivo* [83–86]. Other potential uses include vaccines with overexpression of viral proteins such as rabies virus glycoprotein or cancer therapy [87–89].

### 6.1. Neurodegenerative diseases

After injection in the brain parenchyma, CAV-2 vectors preferentially target neurons and are efficiently retrograde transported into afferent brain regions. The transduction of the substantia nigra pars compacta following intrastriatal injection clearly suggests that these vectors may be well suited for the study and possible therapy of Parkinson’s disease. CAV-2 vectors could be used to modify a panel of gene expression in neurons by using either cell-specific promoters controlling a transgene of interest or encoding siRNAs that could potentially knockout a protein. For instance, one could analyze the effect of localized expression of neurotrophic factors such as ciliary or brain-derived neurotrophic factors, on Alzheimer’s, Huntington’s and many other neurodegenerative disorders.

CAV-2 vectors have been used to study the role of dopamine on fundamental behavior such as movement, feeding, reward responses and learning [83]. Dopamine-deficient (DD) mice were generated to allow a selective restoration of normal dopamine signaling to specific brain regions. These DD floxed stop mice had a non-functional tyrosine hydroxylase (TH) gene due to insertion of a NeoR gene flanked by loxP sites in the first intron of the TH gene. Injection of a CAV-2 vector expressing Cre recombinase in the central caudate putamen restored normal TH gene expression to the midbrain dopamine neurons. Transduced dopaminergic neurons expressed normal cellular proteins (TH) for >1 year post-transduction [83]. In another study, early-born mouse neurons could i) be transduced by CAV-2 vectors in organotypic slices, ii) migrate to distal regions, iii) display a voltagegated sodium current, iv) express functional receptors, and v) show postsynaptic events. These data demonstrated that E1-deleted CAV-2 vector internalization do not significantly disrupt the normal physiology of differentiating neural progenitor cells [84].

Recently, CAV-2 vectors have been tested for their ability to improve neuropathological changes associated with the lysosomal storage disorder, mucopolysaccharidsosis (MPS) type IIIA. MPS’s are a group of lysosomal storage disorders that arise from deficiencies in the catabolism of glycosaminoglycans. At present, there are 11 known MPS, each resulting from the delivery of a different lysosomal enzyme. Of the MPS, MPS IIIA is one of the most common, and as far as treatment goes, one of the most intractable with symptoms including neurocognitive decline, hyperactivity and aggressive behavior. Direct injection of recombinant N-sulfoglucosamine sulfohydrolase (SGSH) into the brain parenchyma or cerebrospinal fluid of MPS IIIA mice improved many of the neuropathological features of the disease [90,91]. However, one of the major limitations of enzyme replacement therapy is the requirement for repeated administration. In contrast, an alternative strategy such as gene therapy is likely to generate persistent transgene expression. In MPS I, III and VII, the respective lysosomal enzymes can be secreted, then internalized by neighboring cells using the mannose-6-phosphate receptor. Because of this phenomenon, the number of cells that need to be genetically modified may be low but scattered throughout the brain. This pathophysiology is the reason CAV-2 vectors are particularly attractive. Recently, an E1-deleted CAV-2 vector expressing SGSH was intracerebrally injected in MPS IIIA mice [86]. Neonatal administration produced both dose-dependent and wide-spread transgene expression which persisted for at least 20 weeks and was sufficient to normalize memory and learning deficits in these mice. In contrast, injection of the same vector into the thalamus and ventricles of adult MPS IIIA mouse brain resulted in dose-dependent, but relatively short-lived gene expression. This loss of expression was likely due to the efficient transduction of ependymal cells in the ventricles during stereotaxic injections. Ependymal cells are efficient antigen presenting cells that, in turn, induced immune response against transduced cells. Besides the use of immunosuppressive drugs to improve the duration of transgene expression in adult mice, a more clinically relevant approach would be to use helper-dependent vectors.

### 6.2. Cancer therapy

Oncolytic conditionally-replicating adenoviruses (CRAds) are a class of anti-cancer agents with therapeutic potential [92]. In this approach, viruses selectively replicate in cancer cells and lead to destruction of the infected cells by virus-mediated cytolysis (CRAds are covered in the review by Toth *et al.* in this issue). Briefly, several oncolytic viruses have been created. Many anticancer strategies are based on the well-studied HAd5 system. To date, clinical efficacy observed in human trials has failed to reach the expectations that were based on studies in animal models [93]. This is due in part to the limited efficiency of infection caused by the absence of CAR expression in many cancer (including gastrointestinal cancers, pancreatic cancer, ovarian cancer and hormone-refractory prostate cancer) and difficulties to obtain tumor-specific replication. Moreover, the immunogenicity of HAd serotypes and the abrogation of propagation due to immune response may be other reasons for the lower than expected clinical efficacy. Finally, complete tumor eradication needs an efficient dissemination of progeny virions in the tumor. This lateral spread of virus appeared limited by both cellular and tissue barriers between neighboring cancer cells [94]. In summary, improving the oncolytic potency of these viruses has been hampered by the inability to study host-vector interactions in immune-competent systems. Notably, HAd do not productively replicate in animal tissues, with some exceptions like Syrian hamsters [95]. Therefore, approaches such as immunomodulation, which could result in sustained replication and subsequently increased oncolysis, are of particular interest.

For these purposes and to treat “man’s best friend”, a CAV-2 conditionally replicating vector was created which effectively replicates in and causes oncolysis of canine osteosarcoma cells. In this vector, the E1A expression was driven by an osteocalcin promoter that restricts its expression and therefore CAV-2 propagation to such osteosarcoma cells [89]. Canine osteosarcoma is the most common primary bone tumor of dogs and is an interesting model of human counterpart. This virus effectively replicated and killed canine osteosarcoma cells *in vitro*. The next step was to test the efficiency and the toxicity of such vector in normal dogs before potential clinical trials in dogs [96]. Short-term physiologic indicators of stress and shock, as well as gross and histological changes in a variety of tissues were analyzed and no major signs of virus-associated toxicity were noted. Interestingly, short-term immunosuppression, allowing increased adenoviral gene transfer and reduced T-cell mediated and neutralizing antibody formation, did not increase CRAd toxicity. These results are particularly pertinent for translation into human Phase I trials.

### 6.3. Vaccine vectors

Due to their aptitude to induce potent innate and adoptive immune responses, Ad vectors have been and are being explored as vaccine carriers. Until recently, the most promising vaccines were based on E1 or E1/E3 deleted HAd5 vectors. In preclinical models, these vectors induced potent transgene product-specific T- and B-cell responses [97]. However, pre-existing neutralizing antibodies to HAd5 virus drastically reduce vector efficacy in animals [98] as well as in human [99,100]. Specific CD8+ T lymphocytes eliminated vector-transduced cells and thus shortening the duration of antigen expression.

Interestingly, the detection of high frequencies of HIV-1-specific CD4 and CD8 T cells in HIV-1-infected subjects with nonprogressive disease [101] and the demonstration that CD8 T cells are key players *in vivo* in the control of SIV replication [102] provided the rationale for developing T-cell vaccine strategies against HIV-1. Expressing conserved antigens of HIV-1 for induction of CD8+ T-cell responses, early phase clinical trials with HAd5 vaccine yielded sufficiently promising results in volunteers at low risk for HIV-1 acquisition to allow for a large phase 2b trial in human at high risk for HIV-1 infection. This STEP trial was prematurely stopped because an interim analysis showed lack of efficiency and even more a twofold increase in the incidence of HIV acquisition among vaccinated recipients with high HAd5 neutralizing antibodies titers compared with placebo recipients [103]. In a recent study, we suggested that this increase in acquisition of HIV infection could be due to the presence of high HAd5 NAbs titers favoring the formation of HAd5 immune complexes (IC) [82]. Compared to HAd5 vector alone, HAd5 IC induced “hyperactivation” and maturation of DCs and therefore a larger expansion of both HAd5-specific memory CD4 and CD8 T cells. Expansion prevent effective generation of the primary immune response against the vector-encoded HIV Ags through notably the killing of DCs and enlarge the pool of memory CD4 T cells that effectively support HIV replication and spreading, thus facilitating susceptibility to HIV infection.

To circumvent problems caused by the pre-existing vector immunity, an alternative approach is the use of vectors derived from nonhuman Ad types. Various nonhuman Ad are currently under investigation as gene expression and vaccine vectors such as CAV-2 but also bovine Ad serotype 3 (BAd3), chimpanzee Ad serotype 1, ovine Ad serotype 7 (OAd7), porcine Ad serotype 3 and 5 (PAd3, PAd5) and fowl Ad serotype 1, 8, 9 and 10 [20,22,104–108].

A very effective vaccine based on an avirulent CAV-2 vector is used worldwide for the routine vaccination against both canine infectious hepatitis (CAV-1) and canine infectious laryngotracheitis (CAV-2), with an excellent safety record. Interestingly, this vaccine is usually administered subcutaneously but it is also effective when administered mucosally [109]. More recently, a replication-competent recombinant CAV-2 vaccine was constructed by homologous recombination with canine distemper virus (CDV) antigen replacing the CAV-2 genomic E3 region [110]. Like in HAd5, the E3 region was non-essential for viral replication in tissue culture. This recombinant vaccine stimulated a protective response in both dogs and minks [110].

Recently, a similar strategy was used to develop a recombinant vaccine in which the glycoprotein gene of rabies (CVS) was cloned and expressed. The effectiveness in protecting against CVS was demonstrated in dogs, cats and pigs [87,88,111].

Importantly, like most other adenoviruses, CAV-2 is also prevalent in their natural hosts and neutralizing antibodies against CAV-2 can have a negative effect on vaccine efficiency. For instance, intranasal vaccination with CAV-2 vector expressing CDV hemaglutinin induced significant levels of CDV-specific immunity in seronegative puppies but a poor immune response in the puppies preexposed to CAV-2 [110]. This difference was attributed to the presence of an anti-CAV-2 mucosal immunity in these pre-exposed animals. As mentioned above, neutralizing antibodies against CAV-2 are not prevalent in humans and are not cross-neutralized by HAd neutralizing antibodies. Therefore, CAV-2 vectors may be an alternative for human as well as veterinary use.

## Conclusion

Significant progress has been made in the understanding of the biology of CAV-2 and in particular CAV-2 vectors. The paucity of pre-existing immunity in human, its preferential interaction with CAR and therefore a neuronal tropism in the CNS, and its axonal transport are characteristics favorable to address fundamental neurobiological questions and to develop potential treatment of neurodegenerative disorders. Currently, CAV-2 vectors have found a niche in gene transfer to the CNS, which limits it to labs with expertise in stereotactic brain surgery and interest in gene transfer. In addition to the native tropism of CAV-2 vectors, mutant vectors harboring a point mutation that abolishes CAR interaction, would be an ideal platform for targeted gene delivery for numerous tissues.
